# Endoscopic rescue after lumen-apposing metal stent deployment failure during endoscopic ultrasound-guided drainage of a giant pancreatic pseudocyst

**DOI:** 10.1055/a-2771-4485

**Published:** 2026-01-28

**Authors:** Meiru Liu, Ziyu Liu, Huihong Zhai

**Affiliations:** 1 71044 Department of Gastroenterology, Xuanwu Hospital, Capital Medical University, Beijing, China

A 32-year-old man with a 1-year history of acute pancreatitis presented with obstructive jaundice caused by a large pancreatic body–tail pseudocyst (14.5 × 6.6 × 10.0 cm) compressing the bile duct ( Fig. 1 ).

**Fig. 1:**
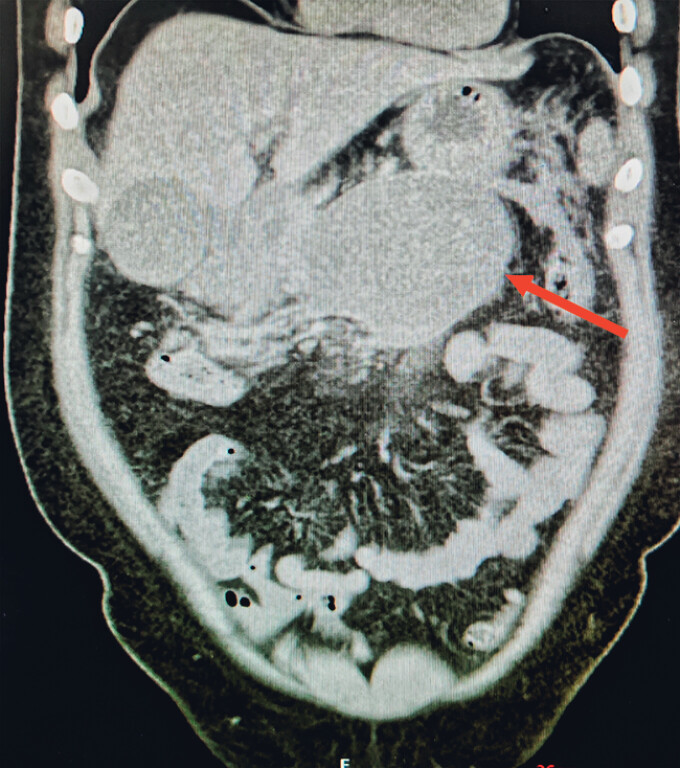
A pseudocyst located in the body–tail region of the pancreas.

During endoscopic ultrasound-guided HOT-AXIOS lumen-apposing metal stent (LAMS) drainage, proximal flange deployment failed and the stent migrated into the pseudocyst ( Fig. 2 **a, b** ). As the original tract was not identifiable, the pseudocyst was punctured via the gastric wall with a 19-G needle, a guidewire was advanced, and the tract dilated to 12 mm. Due to an unfavorable angle, endoscopic entry failed, so two 7 F × 7cm double-pigtail stents were placed for temporary drainage ( Fig. 2 **c** ).

**Fig. 2:**
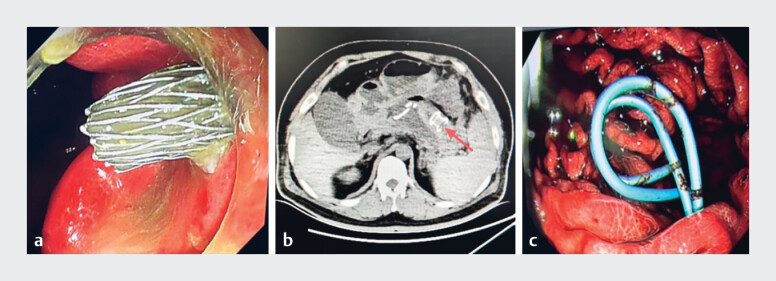
**a** Failed deployment of the proximal flange of the LAMS. **b** Migration of the LAMS into the pseudocyst cavity. **c** Placement of double-pigtail stents. LAMS, lumen-apposing metal stent.

The next day, a salvage procedure was performed. Despite right lateral positioning, direct access remained difficult. The tract was dilated with an 18-mm balloon and further enlarged with an insulation-tipped (IT) knife ( Fig. 3 **a, b** ). After removing the pigtail stents, the endoscope entered the pseudocyst, the migrated LAMS was retrieved with forceps ( Fig. 4 **a, b** ), and a nasocystic tube was placed for drainage ( Video 1 ).

**Fig. 3:**
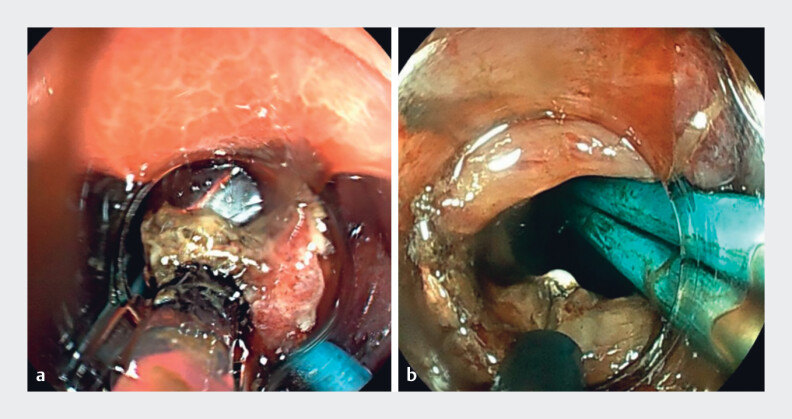
**a** Endoscopic 18-mm balloon dilation. **b** Endoscopic incision and enlargement using an IT knife. IT, insulation-tipped.

**Fig. 4:**
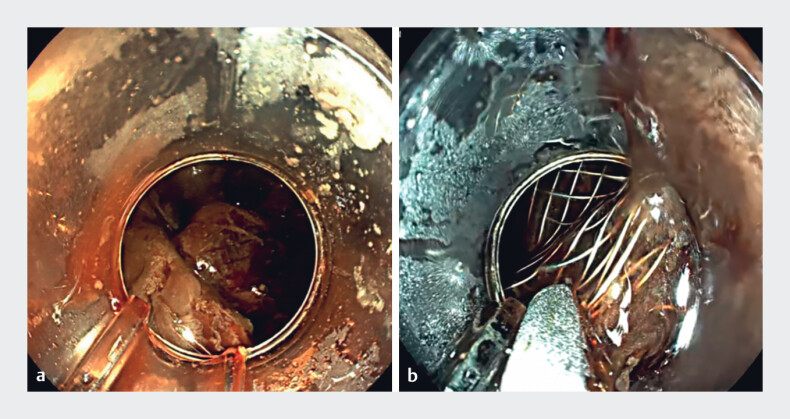
**a** LAMS within the pseudocyst. **b** The LAMS was retrieved from the pseudocyst cavity using grasping forceps. LAMS, lumen-apposing metal stent.

Endoscopic tract dilation with an 18-mm balloon and IT-knife incision was performed to retrieve the migrated LAMS. IT, insulation-tipped; LAMS, lumen-apposing metal stent.Video 1

Two weeks later, endoscopy showed a narrowed tract with persistent drainage, and computed tomography confirmed significant pseudocyst reduction ( Fig. 5 ).

**Fig. 5:**
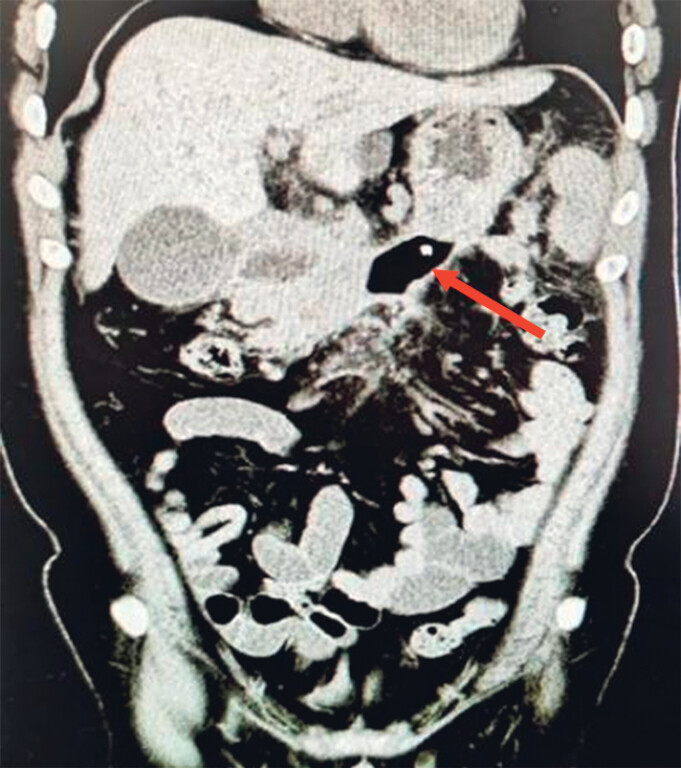
Marked reduction of the pseudocyst 2 weeks after the procedure.

Stent deployment failure is a challenging complication in endoscopic drainage of pancreatic pseudocysts 1 . Endoscopic salvage provides a feasible treatment option 2 , and combined IT-knife incision with balloon dilation allows safe, controlled pseudocyst access, offering a key technical approach for managing LAMS deployment failure.

Endoscopy_UCTN_Code_CPL_1AL_2AD

## References

[1] Pausawasdi N Rugivarodom M Rujirachun P Effectiveness and Safety of a Single 7-French Plastic Stent for Endoscopic Ultrasound-guided Pancreatic Pseudocyst Drainage and Long-term Follow-up Outcomes J Med Ultrasound 2021 29 250 257 10.4103/JMU.JMU_148_20 35127404 PMC8772472

[2] Cahen D Rauws E Fockens P Endoscopic drainage of pancreatic pseudocysts: long-term outcome and procedural factors associated with safe and successful treatment Endoscopy 2005 37 977 983 16189770 10.1055/s-2005-870336

